# Is Oral Testosterone the New Frontier of Testosterone Replacement Therapy?

**DOI:** 10.7759/cureus.27796

**Published:** 2022-08-08

**Authors:** Syed W Ahmad, Gianfranco Molfetto, David Montoya, Ariday Camero

**Affiliations:** 1 Hospital Medicine, Nova Southeastern University Dr. Kiran C. Patel College of Osteopathic Medicine, Fort Lauderdale, USA; 2 Medical Education and Simulation, Nova Southeastern University Dr. Kiran C. Patel College of Osteopathic Medicine, Fort Lauderdale, USA; 3 Medicine, Florida International University, Miami, USA

**Keywords:** trt, male hypogonadism, oral testosterone, im testosterone, transdermal testosterone

## Abstract

Male hypogonadism is a condition in which the body does not produce enough testosterone, resulting in symptoms such as depressed mood, decreased sex drive, decreased skeletal muscle, and increased fat mass. Male hypogonadism can be readily treated with many available treatments when clinically indicated. The advent of readily available testosterone therapy has increased the importance of finding the most efficacious and cost-efficient treatment modality to approach these patients. Testosterone is typically administered through intramuscular or subcutaneous injections, topical gels, and oral tablets. The efficacy of testosterone therapy on hypogonadal men has been widely studied. However, there has been little research done comparing each modality against each other. This paper seeks to compare the various modalities of testosterone replacement therapy using various parameters such as the beneficial effects on bone mineral density, skeletal muscle mass, fat mass, and libido while simultaneously weighing the distinct undesirable side effects of each form of administration. Our investigation analyzes the methodology and results of the existing research within this field. It aims to draw a nuanced conclusion about the current standard of care for testosterone replacement therapy. According to our research and statistical analyses, we have concluded that oral administration has shown to be as advantageous as other modalities for male hypogonadism. Currently, injectables are the modality of choice, but with the right improvements, oral administration can potentially overtake injectables and transdermal testosterone as the treatment of choice.

## Introduction and background

Male hypogonadism is characterized by a deficiency in the body’s innate ability to produce testosterone, usually due to some dysfunction of the hypothalamic-pituitary-gonadal axis. The characteristic sequela of testosterone deficiency is categorized by symptoms such as decreased libido and mood and signs such as decreased serum testosterone levels and a change in body composition. Changes in body composition are associated with both a decrease in skeletal muscle mass and an increase in fat mass. The use of testosterone therapy in patients with androgen deficiency syndromes has unequivocally been proven to benefit in all the parameters mentioned above [1]. Testosterone administration for the treatment of hypogonadism routinely consists of either transdermal application of specially formulated gels, intramuscular/subcutaneous injections, or oral tablets taken by mouth. Recently, the United States FDA approved the use of oral testosterone undecanoate in treating men with certain forms of hypogonadism.

The American Urological Association recommends treating patients on a case-by-case basis utilizing a combination of signs and symptoms and serum testosterone levels. According to Paduch et al., there is no “universally accepted threshold of Testosterone (T) concentration that distinguishes eugonadal from hypogonadal men” and, therefore, no current standard of care guidelines for the treatment of hypogonadism [2]. However, there are European guidelines set forth by the Endocrine Society that currently recommend 75-100mg of testosterone enanthate or cypionate administered via intramuscular injections weekly or 150-200mg administered every two weeks. One or two 5mg non-genital testosterone patches can be applied nightly over the skin of the back, thigh, or upper arm (away from pressure areas). 5-10g of a 1% testosterone gel applied daily over a covered area of non-genital skin. 30 mg of a bioadhesive buccal testosterone tablet can be applied to buccal mucosa every 12hr. Testosterone pellets are implanted subcutaneously (SC) at intervals of 3-6 months, with the dose and regimen varying with the formulation used. Lastly, oral testosterone undecanoate, injectable testosterone undecanoate, testosterone-in-adhesive matrix patch, and testosterone pellets where available [3]. This review summarizes the current strengths and weaknesses of oral, injectable, and transdermal testosterone administration and highlights the possible advancements in testosterone therapy in the United States.

## Review

Materials & Methods

We searched ScienceDirect, MEDLINE, and PubMed databases and included publications from January 1st, 1970, to March 1st, 2021. We selected a total of thirty-three articles (including both primary and secondary studies) in which researchers examined the use of injectable, transdermal, and oral testosterone therapy along with its effects on any of the following: serum hormone levels, body composition (including skeletal muscle mass and fat mass), libido, mood, and adverse effects. The studies were then screened for possible therapy effects on serum hormone levels, body composition (including skeletal muscle mass and fat mass), libido, mood, and adverse effects; afterward, relevant data was extracted.

Results

Injectable therapy

The traditional method for delivering androgens to the body is via injection, either intramuscularly or subcutaneously. The testosterone molecule is attached to an ester that allows it to be taken up through the tissue and delivered systemically. Testosterone enanthate and testosterone cypionate formulations are clinically available for use in the US. Both formulations require either weekly or biweekly injections to maintain stable blood levels and prevent fluctuations.

Serum Testosterone Levels

The effects of injectable testosterone on serum total and serum free testosterone have been widely studied. In a study by Behre et al., intramuscular substitution therapy was applied to 52 patients with 250mg testosterone enanthate, injected almost every three weeks. In their study, they were able to determine that testosterone replacement therapy for up to 16 years yielded a normalization of testosterone serum levels in all patients. Baseline levels increased from (164 ±17 ) ng/dL in untreated hypogonadal patients to (551 ± 38) ng/dL in all patients during therapy [4]. Similarly, Bhasin et al. studied the effects of 100mg testosterone enanthate given IM weekly to hypogonadal men. After 10 weeks of treatment, they found that total serum testosterone rose from (72 ± 29) ng/dL to (767 ± 182) ng/dL, and free serum testosterone increased from (19.0 ± 6.9) pg/mL to (69.0 ± 8.1) pg/mL [5].

Body Composition

In Behre et al.’s previously mentioned study they investigated bone mineral density (BMD) changes in hypogonadal men treated with testosterone preparations over a 16-year period. In the 32 hypogonadal men with quantitative computed tomography (QCT) measurements before treatment, multiple regression analysis revealed a significant association of BMD with serum levels of testosterone and with age. Testosterone therapy resulted in an increase in BMD from (95.2 ± 5.9) to (120.0 ± 6.1) mg/cm^3^ after the first year [4]. Amory et al. also evaluated the effects of 200mg testosterone enanthate injected biweekly on the effects of bone mineral density. After 36 months of treatment, the subjects were measured to have a (1.06 ± 0.16)% increase in lumbar spine density and a (0.75 ± 0.11)% increase in trochanteric density [6]. In terms of lean body mass, Bhasin et al found that weekly 100mg IM injections of testosterone enanthate on average produced a 5.0 ± 0.7 kg increase after a 10-week period [5]. 

In a study recently published by Saad et. al in February of 2019 two groups of men with hypogonadism were categorized under having no/mild erectile dysfunction and moderate/severe erectile dysfunction (ED). The patients of both groups who chose to accept testosterone therapy (TTh) were administered injections of testosterone undecanoate entirely through intervals of 3-month time periods; to the exception of an initial 6-week interval. Patients would regularly be measured for anthropometric measurements during their routine follow-ups at the practice; the data was averaged across each year. Data at the 10-year mark for follow-ups when estimated to show differences amongst both groups displayed that there was an average decrease of 18.4±0.5 kgs (no/mild ED TTh treated group) and 18.0±0.4 kg (moderate/severe ED TTh treated group). Both groups that were treated with testosterone undecanoate went on to show an average decrease in waist circumference of 10.3±0.3 cms (no/mild ED TTh treated group) and 9.5±0.3 cms (moderate/severe TTh treated group). BMI group averages also showed a drop of 5.8±0.2 kg/m^2^ (no/mild ED TTh treated group) and 5.8±0.1 kg/m^2^ (moderate/severe ED TTh treated group) [7].

Quality of Life

In the previously mentioned study by Saad et al., several quality-of-life factors were recorded to show improvement, including blood pressure, lipid quantity, urinary function, erectile function, and even AMS (aging males' symptoms) scores. Urinary function, erectile function, and AMS are measured through a questionnaire. The questionnaires are designed to assess the effects of erection problems in a person's life (IIEF), assess the symptoms of aging between groups of aging men under different circumstances (AMS), and assess urinary function based on a set of questions concerning urinary function (IPSS). An average drop of 19.4±0.9 mmHg (no/mild ED TTh treated group) and 22.5±0.9 mmHg (moderate/severe ED TTh treated group) was recorded for systolic pressure in both TTh treated groups; an average drop of 11.5±1.0 mmHg (no/mild ED TTh treated group) and 11.1±0.8 mmHg (moderate/severe ED TTh group) for diastolic pressure. Lipid quantity showed an average decrease in both non-HDL cholesterol by 4.1±0.2 mmol/L (no/mild ED TTh treated group) and 4.1±0.2 mmol/L (moderate/severe ED TTh treated group) and triglycerides by 1.0±0.0 mmol/L (no/mild ED TTh treated group) and 0.9±0.0 mmol/L (moderate/severe ED TTh treated group). All the while still showing an average increase in HDL cholesterol by an average of 0.4±0.0 mmol/L (no/mild ED TTh treated group) and 0.3±0.0 mmol/L (moderate/severe ED TTh treated group). Improvements in urinary function were based on scores from the IPSS, which in both TTh treated groups showed a decrease of 4.6±0.2 (no/mild ED TTh treated group) and 4.6±0.2 (moderate/severe ED TTh treated group) [7]. 

In a separate study on injectable testosterone therapy by Schiavi et al., a significant increase in the frequency of ejaculation and sexual desire in those receiving IM testosterone versus placebo was discovered [8]. Additional data from Saad et al. supports this, as in their study, erectile function was also measured, it was measured off IIEF-EF scores, which recorded an average increase in the TTh tested groups of 4.4±0.2 (no/mild ED TTh treated group) and 11.1±0.3 (moderate/severe ED TTh treated group). AMS scores were recorded and showed a decrease of 31.9±0.3 (no/mild ED TTh treated group) and 27.3±0.5 (moderate/severe ED TTh treated group) in the two groups. These AMS and IIEF-EF scores were accompanied and could also be related to improving urinary function in TTh treated groups. Improvements in urinary function were based on scores from the IPSS, which in both TTh treated groups showed a decrease of 4.6±0.2 (no/mild ED TTh treated group) and 4.6±0.2 (moderate/severe ED TTh treated group) [7]. 

Adverse Effects

Bhasin et al. found that AST/ALT levels after 10 weeks of injectable testosterone enanthate therapy were reduced [5]. In a study by Turner et al., patients experienced pain at the injection site up to 24 hours after treatment [9]. There is a correlation between high testosterone levels and high hemoglobin. Erythrocytosis, or polycythemia, is a known side effect of testosterone replacement therapy (TRT). Testosterone injection is associated with a higher potential for erythrocytosis than topical preparations. Fernandez-Balsells et al. found 11 trials highlighting erythrocytosis as a prominent side effect of TRT in hypogonadal men [10]. Another study was able to demonstrate that TRT caused statistically significant increased hemoglobin levels (0.86 ± 0.31g/dl, p = 0.01) [11]. In a study by Raynaud et al., there was an elevated prostate-specific antigen (PSA) level due to IM testosterone over six years [12].

Transdermal therapy

Transdermal testosterone modalities are applied to the skin, which allows for the perpetual release of the hormone at a more regular rate when compared to injectable delivery systems. Gel formulations, such as Androgel or Axiron, contain alcohol and are recommended for application to the epidermis in any area beside the scrotum. The alcohol content allows for rapid dissolution of the gel on the skin. Scrotum application is contraindicated due to increased levels of 5 alpha-reductase, which can subsequently contribute to prostatic hyperplasia due to the conversion of testosterone to 5-DHT [13]. Testosterone can also be delivered through transdermal means with a patch. These patches imitate the diurnal pattern of testosterone release seen in human physiology. Testosterone levels post application peak at 2-6 hours and fall back within 24 hours, thus necessitating renewed application within 24 hours. As with the testosterone gel modality, transdermal patches may be applied anywhere along the skin apart from the scrotum, buttocks, or bony site. It is recommended that the application site should be regularly rotated around once a week to reduce the risk of an adverse dermatological reaction.

Serum Testosterone Levels

Changes in serum testosterone levels with testosterone supplementation are well studied throughout the available literature involving the study of hypogonadism. When using transdermal methods, it typically takes around 4-12 weeks to improve serum testosterone levels to the acceptable range of 400-700 ng/dL [14]. In a prospective, open-label study by J. Rodriguez-Tolra et al., 50 hypogonadal males were given 50mg of testosterone gel daily. Total and free testosterone levels were measured after 12 and 24 months of therapy. Baseline total testosterone for the participants was measured at (294±104)ng/dL and free testosterone was (51.9±14.4) pg/mL. The measurements at 12 months for total and free testosterone were (555±291) ng/dL and (121.2±83.7) pg/mL, respectively. At 24 months the measurements were (553±250) ng/dL total testosterone and (115.4±49.0) pg/mL free testosterone [15]. Wang et al. also conducted a study of 222 subjects in which 73 participants were given 50mg testosterone gel, 78 participants were given 100mg testosterone gel, and 76 were given a testosterone patch daily. The 50mg/day T gel group saw increased total testosterone from (237 ± 15) ng/dL to (555 ± 34) ng/dL after 180 days of treatment. The 100mg/day T gel group saw increased total testosterone from (248 ±16) ng/dL to (713 ± 30) ng/dL after 180 days. The patch group went from (237 ± 16) ng/dL at baseline to (408 ± 25) ng/dL after 180 days [16].

Body Composition

In J. Rodriguez-Tolra et al.'s previously mentioned study, total bone mass density was also measured during months 12 and 24 in the lumbar spine, femur, trochanter, and Ward's triangle using a DEXA scan. The study showed a significant improvement in bone mass density across all the measured areas. At 50mg/day of testosterone gel, the lumbar spine (L1-L4) showed a 4.5% increase in BMD, while the trochanter showed a 3.2% increase. It was also discovered that C-telopeptide decreased, which shows that bone destruction had been decelerated [15]. Measurements of lean body mass and fat mass were recorded by Wang et al., in the T gel 50mg/day group an increase in lean body mass of (1.59 ± 0.39) kg was seen after 180 days. The T gel 100mg/day group increased (3.03 ± 0.35) kg after 180 days. The T patch group had a change of (0.99 ± 0.38) kg after 180 days. In terms of body fat mass, the T gel 50mg/day group saw a decrease of (0.90 ± 0.32) kg, the T gel 100mg/day group saw a decrease of (1.05 ± 0.22) kg, and the T patch group saw no change (0.01 ± 0.2) kg. These results were seen at 90 days and decreased in the T gel groups after 180 days [16].

Quality Of Life

We gathered information regarding the effects of transdermal testosterone on quality-of-life factors, including mood and sexual function. According to a meta-analysis by Pankaj Jain et al., transdermal testosterone therapy was more effective than oral and injectable modalities when looking at their effects on erectile dysfunction. Transdermal patch delivery had a response rate of 80.9% compared to 51.3% achieved by intramuscular delivery and 53.2% achieved by oral supplementation. The authors scoured various databases for 73 articles investigating the effects of testosterone replacement therapy on erectile dysfunction to spur new research. It was suggested that this discrepancy in response rates might be due to the size of the studies; the transdermal study had 42 participants, whereas the intramuscular studies had much larger participation [17]. In a separate meta-analysis by Giovanni Corona et al., a study conducted with more than 700 patients using a transdermal delivery method showed a significant improvement in the patient's sexual function [18].

Adverse Effects

Testosterone gels are more commonly used as treatment modalities for hypogonadism due to their ease of use and patient preference. However, there are serious concerns surrounding their use. Wang et al. found that skin irritation was reported in 5.5% of subjects treated with testosterone gel and 66% of subjects in the testosterone patch group [16]. Also, it is important to note that women and children should avoid contact with clothing or skin that may have gel residue due to reported side effects such as precocious puberty caused by secondary transfer. Cavender et al. performed a case study where such an incident occurred in a 10-month-old male. The patient had developed precocious puberty due to the transfer of testosterone from the patient's father, who had been undergoing treatment for hypogonadism using a topical gel. The patient's symptoms subsided once his father's treatment had been changed to a buccal modality [19]. Another case study by Brachet and Heinrichs discovered that a 5-year-old patient had developed central precocious puberty after long-term exposure to testosterone via secondary interpersonal transfer from testosterone gel, as determined by a GnRH test. The patient's secondary exposure had begun while he was in utero [20]. When considering transdermal gels there is a deep concern for transference through contact that can lead to virilization, especially in children [21]. Certain transdermal gels do not allow the patient to wash the application site for hours, increasing the probability of transfer [22]. No significant changes in liver function tests were noted in the previously mentioned Wang et al. study comparing T gel uses of 50 mg, 100 mg, and T patch; however, measured PSA levels throughout treatment did show significant changes. The study noted a significant increase in mean serum PSA levels in the 100 mg T gel group, going from (0.89±0.08) ng/mL upon the initial measurement on day 0 to (1.19±0.12) ng/mL on day 90. The 50 mg/day group demonstrated a baseline PSA level of (0.88±0.08) ng/mL which increased to (1.19±0.12) ng/mL. There was no significant change to PSA levels noted in the T patch group; baseline levels were measured to be (0.89±0.10) ng/mL, and on day 90, the PSA levels were (0.88±0.09) ng/mL [16].

Oral therapy

Androgens traditionally cannot be given orally due to poor bioavailability from extensive hepatic first-pass metabolism [23]. The only ester testosterone preparation available for administration by mouth is testosterone undecanoate. The greatest absorption of testosterone undecanoate occurs with the simultaneous consumption of meals high in fat concentrations. This is due to a "hydrophobic, long aliphatic chain ester," which "favors preferential absorption into chylomicrons entering the gastrointestinal lymphatics and largely bypassing hepatic first-pass metabolism." Despite adding an ester, multiple high daily dose regimens of 160 mg or 240 mg are deemed most effective due to undecanoate's variable and inconsistent bioavailability and short duration of action [24]. After compiling studies that were done regarding oral testosterone therapy, the following results were determined:

Serum Testosterone Levels

In a single-blind study by Zhang et al., 160 men at least 50 years of age presented with a total morning serum testosterone measurement of < 230 ng/dL or a morning free testosterone level of <64.9 pg/mL. If the serum total morning testosterone level was between 230 and 345 ng/dL, participants were placed in either a treatment or placebo group. The treatment group received 120-160 mg of testosterone undecanoate (TU) oral daily, based on serum testosterone levels, while the placebo group received vitamin e/c capsules. The researchers found that serum total testosterone concentrations before and after intervention were (230 ±21) ng/L and (395±34) ng/dL, respectively, and the free testosterone concentrations before and after intervention were (30.0±5.0) pg/mL and (62.1±9.0) pg/mL [25]. Mean total serum testosterone and free testosterone concentrations of all patients at six-month follow-up remained within the reference range of adult males and were significantly higher than the corresponding baseline levels [25].

Park et al. conducted a similar study by administering oral testosterone undecanoate in a single-blind, placebo-controlled study to 33 participants with hypogonadism. Patients were selected using a similar criterion to the above study; 27 were given 80mg of testosterone undecanoate twice a day, while six were given placebos. In regards to serum total testosterone levels, there was an increase from (260±130) ng/dL to (400±180) ng/dL after three months of therapy, which was deemed statistically significant [26]. In a recent open-label study conducted by Swerdloff et al., 221 male patients between 18-65 years of age with consistent serum total T <300 ng/dL and a history of hypogonadism-like signs or symptoms were randomly assigned to either an oral testosterone undecanoate group or a 2% topical testosterone solution group. The oral testosterone undecanoate group started the study with a dose of 237 mg of testosterone undecanoate twice a day prior to breakfast and dinner to allow for a 12-hour window. Based on average testosterone levels measured on days 21 and 56 of the study, doses could be changed to 316 mg and then 396 mg or decreased to 198mg and 158mg of testosterone undecanoate. At the end of the 105-day study, 87.3% of the oral testosterone undecanoate group patients displayed serum testosterone levels of 489±128 ng/dL [27]. From these findings, it is safe to conclude that regarding serum testosterone levels, administering testosterone undecanoate orally is an effective method in bringing patients with hypogonadism to therapeutic physiological levels.

Body Composition

Changes in bone mineral density, skeletal muscle mass, and fat mass while receiving oral testosterone therapy have been well acknowledged. Bouloux et al. found that oral testosterone undecanoate significantly increased bone mineral density in the lumbar spine (L1-L4) and the trochanter at 160 mg/d compared with placebo. After 12 months of therapy, there was a (1.68 ± 3.35) % change in the lumbar spine and a (1.37 ± 4.00)% change in the trochanter. They also reported a dose-dependent response on both lean and body fat mass; 160 mg/day of oral TU resulted in a 1.3 kg increase in lean body mass, while 240 mg/day resulted in a 1.7 kg increase. Body fat mass was reduced by 1.4 and 1.2 kg after 12 months of treatment with oral TU 160 and 240 mg/d, respectively [28]. A study by Wittert et al. highlighted the efficacy of oral testosterone on body composition. After six months of therapy consisting of 160mg/day of oral TU, lean body mass decreased by (0.91 ± 0.03) kg in the placebo group and increased by (1.04 ± 0.07) kg in the testosterone group. Fat mass increased by (0.85 ± 0.19) kg in the placebo group and decreased by (0.2 ± 0.1) kg in the testosterone group [29]. 

*Quality of Life* 

Furthermore, we found that quality of life factors such as sexual function, mood, and mental status was well documented with oral therapy. The same study by Park et al. found that testosterone undecanoate treatment significantly improved sexual dysfunction compared to baseline and placebo. Those in the treatment group reported increased libido, decreased erectile dysfunction, and decreased ejaculation difficulty. Likewise, in terms of mood and mental status, the treatment group, on average, reported significantly decreased nervousness or depression, increased appetite, and increased memory and concentration [26]. Similarly, a study done by Haren et al. on the effects of administering testosterone undecanoate for 12 months on older men with hypogonadism revealed significant improvements in quality of life; the treatment group reported decreased sadness/grumpiness and improved erection strength [30]. In the study conducted by Swerdloff et al., patients included in the oral therapy group were noted to have a significant decrease in negative moods and significant increases in positive moods, sexual desire, weekly sexual activity (with and without a partner), and sexual energy with a partner [27]. The overall quality of life increased in male patients across multiple studies.

Adverse Effects

The general adverse effects of testosterone supplementation have been widely studied, but there has been varying data on oral testosterone itself. Oral testosterone can be prepared in many ways to have reliable system absorption. Methyltestosterone, 17-alkylated testosterone, is absorbed through the portal system and can result in hepatotoxicity. However, testosterone undecanoate is a non-alkylated testosterone ester absorbed through the intestinal lymphatic system and bypasses the portal system, allowing for little to no hepatotoxicity. Park et al. recorded normal AST and ALT levels with TU usage showing a lack of any undesirable effect on the liver.

Similarly, they found no abnormal elevation of PSA or difficulty voiding in the treated subjects [26]. Prior studies have also reported no significant increase in prostate volume, PSA elevation, or new onset of obstructive voiding [31-33]. General side effects of oral testosterone undecanoate therapy have been regarded as "mild" and "self-limiting" [26]. However, there have been documented reports of gastrointestinal intolerance, increased hematocrit, increased hypertension, and decreased HDL cholesterol levels which changed when increasing dosage [24,27,29]. Despite these few accounts of adverse effects, of which some can be quelled by a consistent dosing routine of 80 mg BID [30], oral testosterone undecanoate therapy is considered a very well-tolerated modality.

## Conclusions

Our research has shown that the modalities discussed have nearly equal efficacy in raising serum testosterone levels to be physiologic and therapeutic eugonadal levels. They also have shown similar efficacy in improving body composition and quality of life in mood and sexual function measures. Therefore, each modality's prescription rests on each side effect profile. The major complaint by patients participating in IM testosterone therapy is the pain at the injection site resulting in decreased patient compliance. Transdermal testosterone therapy, such as gels and patches, avoids this pain issue at an injection site and even has the benefit of reducing the number of clinic visits a patient must make; however, they pose other potential severe consequences and side effects. Oral testosterone undecanoate has been found as an equivalent alternative to gel therapy throughout the world, including in Europe; we have found that oral testosterone undecanoate has minimal side effects, which can be quelled by changing the dosage. Ease of use when considering either gels or orals allow for higher patient compliance. However, compared to orals, gels possess the risk of interpersonal testosterone transfer, as noted in various case studies, and increases in PSA levels not seen in the oral treatment. Based on recent approval by the FDA, we believe that more research should be done concerning oral testosterone use for hypogonadal treatment in the US due to its equal efficacy compared to the other modalities, its ease of use leading to increased patient compliance, and its mild side effect profile.
